# Estrogen receptor alpha drives proliferation in PTEN-deficient prostate carcinoma by stimulating survival signaling, MYC expression and altering glucose sensitivity

**DOI:** 10.18632/oncotarget.2820

**Published:** 2014-11-26

**Authors:** Itsuhiro Takizawa, Mitchell G. Lawrence, Preetika Balanathan, Richard Rebello, Helen B. Pearson, Elika Garg, John Pedersen, Normand Pouliot, Robert Nadon, Matthew J. Watt, Renea A. Taylor, Patrick Humbert, Ivan Topisirovic, Ola Larsson, Gail P. Risbridger, Luc Furic

**Affiliations:** ^1^ Department of Anatomy and Developmental Biology, Monash University, Clayton, Victoria, Australia; ^2^ Peter MacCallum Cancer Centre, Melbourne, Victoria, Australia; ^3^ Sir Peter MacCallum Department of Oncology, Melbourne University, Victoria, Australia; ^4^ Lady Davis Institute for Medical Research, Sir Mortimer B. Davis-Jewish General Hospital and Departments of Oncology, Experimental Medicine and Biochemistry, McGill University, Montréal, Canada; ^5^ Department of Human Genetics, McGill University, Montréal, Canada; ^6^ TissuPath Pathology, Melbourne, Victoria, Australia; ^7^ Department of Physiology, Monash University, Clayton, Victoria, Australia; ^8^ Department of Pathology and Department of Biochemistry and Molecular Biology, the University of Melbourne, Parkville, Australia; ^9^ Department of Oncology-Pathology, Karolinska Institutet, Stockholm, Sweden

**Keywords:** Estrogen receptor alpha, prostate cancer, PTEN, metabolism, MYC

## Abstract

While high doses of estrogen, in combination with androgens, can initiate prostate cancer (PCa) via activation of the estrogen receptor α (ERα), the role of ERα in PCa cells within established tumors is largely unknown. Here we show that expression of ERα is increased in high grade human PCa. Similarly, ERα is elevated in mouse models of aggressive PCa driven by MYC overexpression or deletion of PTEN. Within the prostate of PTEN-deficient mice, there is a progressive pattern of ERα expression: low in benign glands, moderate in tumors within the dorsal, lateral and ventral lobes, and high in tumors within the anterior prostate. This expression significantly correlates with the proliferation marker Ki67. Furthermore, *in vitro* knockdown of ERα in cells derived from PTEN-deficient tumors causes a significant and sustained decrease in proliferation. Depletion of ERα also reduces the activity of the PI3K and MAPK pathways, both downstream targets of non-genomic ERα action. Finally, ERα knockdown reduces the levels of the MYC protein and lowers the sensitivity of cellular proliferation to glucose withdrawal, which correlates with decreased expression of the glucose transporter GLUT1. Collectively, these results demonstrate that ERα orchestrates proliferation and metabolism to promote the neoplastic growth of PCa cells.

## INTRODUCTION

Prostate cancer is the second leading cause of cancer-related death in US men; 1 in 7 men will be diagnosed in their lifetime and 1 in 37 will die from prostate cancer [1]. Androgens are the main regulators of prostatic cell proliferation in development and homeostasis. Androgens and the androgen receptor (AR) also drive the progression of prostate cancer, although the disease is often diagnosed at an age when the serum level of testosterone in men is declining [2]. Nevertheless, prostate cancer is an endocrine-related cancer where androgens are necessary, but insufficient, to cause cancer development and progression [3]. Yet, combined treatment of mice or benign human xenografts with high levels of testosterone and estradiol induces prostate carcinogenesis [4, 5]. This demonstrates that along with androgens, estrogens have a critical role in prostate cancer.

Estrogens regulate proliferation and cell death in the prostate and have dual roles in prostate cancer, either pro- or anti-tumorigenic. These specific actions are mediated by the two estrogen receptor (ER) subtypes, ERα or ERβ [6]. The anti-tumorigenic role of ERβ has been studied in detail [7]. Specific activation of ERβ with selective agonists causes apoptosis, decreases tumor growth *in vivo* and inhibits epithelial-to-mesenchymal transition [8-10]. The pro-tumorigenic actions of ERα are comparatively ill-defined. One explanation for the paucity of research into ERα in prostate cancer is the uncertainty about its expression profile; ERα is abundantly expressed in normal and tumor stroma, but there are differing reports about whether it is expressed in epithelial prostate cancer cells [11-17]. Most studies have detected negligible ERα staining in prostate cancer cells using immunohistochemistry. This could be due to an under-representation of high grade samples because other studies showed that ERα expression in prostate cancer cells is significantly associated with high Gleason score and poor patient survival [11, 17]. A second reason that the role of ERα in prostate cancer cells has been underestimated, is that most human prostate cancer cell lines express little or no ERα [18-22]. Therefore, the goal of this study was to resolve the expression profile of ERα in prostate cancer cells and identify new models to study its effects. By focusing on the autonomous role of ERα in prostate cancer cells, including interactions with other signalling pathways, we aimed to provide a more comprehensive understanding of estrogen action in prostate cancer.

The role of ERα in cancer cell proliferation and survival has been extensively studied in the breast and other female tissues such as the ovary, uterus and cervix. These studies demonstrate that ERα is a multifunctional protein; in addition to its genomic role as a transcription factor, where agonist-bound ERα regulates the expression of downstream genes containing estrogen-response elements (ERE) [23], ERα localizes to the cell membrane via palmitoylation to direct “non-genomic” activities [24]. At the plasma membrane, ERα interacts with c-Src and the regulatory subunit of PI3K, p85α, to augment signaling activity through the MAPK and PI3K pathways [25-27], both drivers of growth, proliferation and survival.

The interaction of ERα and survival signaling cascades is potentially significant because they are commonly dysregulated in prostate cancer. The PTEN tumor suppressor is frequently lost through single or bi-allelic deletion, mutations and epigenetic silencing [28, 29]. PTEN insufficiency promotes hyperactivation of the kinase AKT, leading to pro-survival and pro-proliferative signaling. Moreover, loss of PTEN synergizes with loss of p53, over-expression of MYC and sustained activation of MAPK in promoting prostate cancer initiation and progression [30-32]. In our studies of mice with conditional PTEN knockout in the prostate epithelium (*PBiCre^+/−^;Pten^fl/fl^*), we noted a marked up-regulation of ERα expression during cancer progression. This led us to investigate estrogen signaling in the PTEN-deficient model.

Here we show, in the PTEN-deficient model of prostate cancer, that the expression of ERα (but not AR or ERβ) is significantly increased in regions of tumors where cell proliferation is notably enhanced. In addition, in human prostate cancer specimens we demonstrate that ERα expression is associated with regions of high Gleason score, indicative of more aggressive disease. Stable down-regulation of ERα expression in cell lines derived from primary PTEN-deficient tumors induced a striking and sustained reduction in proliferation. Our results indicate that ERα promotes the proliferation in PTEN-deficient tumors by regulating MYC expression and glucose sensitivity.

## RESULTS

### ERα is expressed in prostate cancer cells

**Figure 1:**
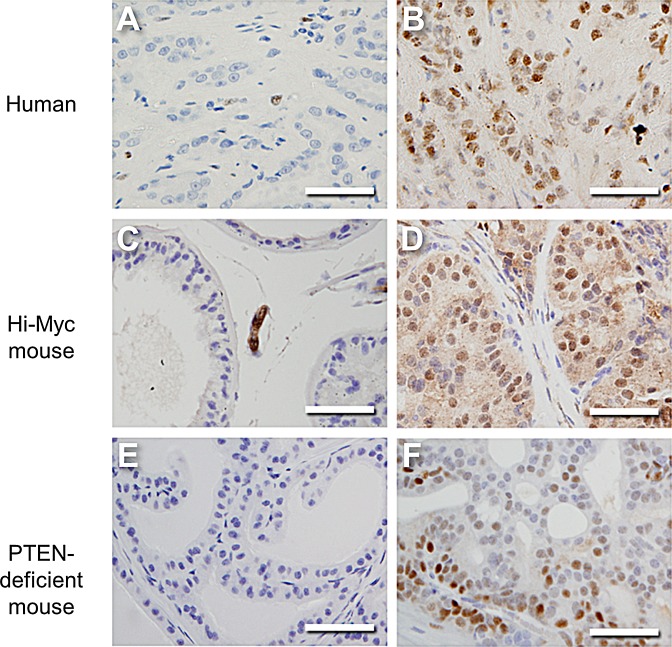
ERα expression increases in prostate cancer Representative images of ERα staining in human and mouse prostate cancer. Specimens of (A) Gleason score 6 and (B) Gleason score 9 human prostate cancer. (C) Benign lateral prostate tissue of a 5 month old control mouse (FVB) compared to (D) prostate cancer in an age-matched Hi-MYC mouse (ARR2/Pbsn-*Myc*). (E) Benign anterior prostate tissue from a 80-100 day old control mouse (*PBiCre*^+/−^) compared to (F) malignant tissue in an age-matched PTEN-deficient animal (*PBiCre*^+/−^*;Pten*^fl/fl^). Scale bars equal 50 μm.

It is contentious whether ERα is expressed in prostate cancer cells or only the stroma [11-13, 17, 22]. This prompted us to re-examine ERα expression in prostate cancer using immunohistochemistry. It has been suggested that ERα is up-regulated in high grade disease [11], so we selected a cohort of Gleason score 9 specimens and a similar number of low grade Gleason score 6 tumors for comparison. As expected, AR was abundantly expressed in the epithelium in all samples (Supplementary Fig. 1 and Supplementary Table 1). ERα was expressed in 48% (10/21) of Gleason score 9 tumors, but no Gleason score 6 tumors (0/15), consistent with a previous study (Fig. 1A and B) [11]. This increase in ERα expression in high grade human prostate cancer was statistically significant (*P*=0.001). In the positive specimens, 10-80% (average 40%) of all cancer cells exhibited prominent nuclear staining of ERα (Supplementary Table 1). These findings warranted further studies into the biological significance of ERα in prostate cancer. Most human epithelial prostate cancer cell lines, however, express little or no ERα, so are inappropriate to study the autonomous role of ERα [18-22]. To identify systems to study the biological significance of ERα, we therefore examined ERα expression in mouse models of aggressive prostate cancer. Consistent with data from our human specimens, ERα levels are increased in tumors from both Hi-MYC (Fig. 1D) and PTEN-deficient (Fig. 1F) mice when compared to normal prostate tissue from control mice (Fig. 1C and E). Collectively, these data show that ERα is expressed in epithelial cells in a subset of aggressive prostate cancers and indicate that a number of mouse models mirror this expression pattern.

### ERα expression correlates with Ki67 in PTEN-deficient murine prostate cancer

**Figure 2:**
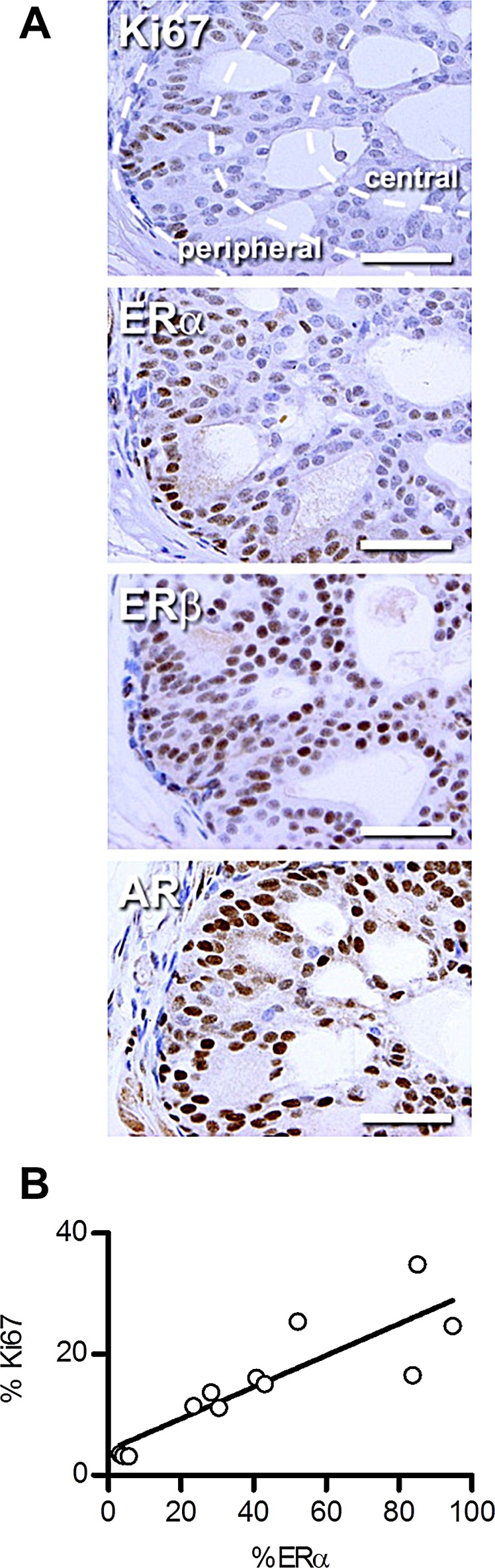
ERα expression correlates with proliferation in PTEN-deficient prostate cancer (A) Representative images of immunohistochemical staining of Ki67, ERα, ERβ and AR in the anterior prostate of 12-14 week old PTEN-deficient mice. The peripheral and central regions of the foci are shown in the Ki67 image. Scale bars = 50 μm. (B) Dot plot comparing the percentage of ERα and Ki67 positive cells in the prostate of PTEN-deficient mice (*P*<0.001, r^2^=0.75, Pearson correlation, n=12 regions from 3 mice).

Further analysis of ERα immunoreactivity in the prostates of PTEN-deficient mice revealed particularly intense staining towards the periphery of the malignant foci. This resembled the band of Ki67-positive proliferating cells that has previously been reported [33] and prompted us to investigate whether ERα and Ki67 levels correlate. To test this, staining was quantified in serial sections from four different zones of the prostate that have distinctive patterns of Ki67 expression (Supplementary Fig. 2A). The highest percentage of Ki67 and ERα positive cells was in the peripheral region of anterior prostate (AP) tumors (Fig. 2A). In contrast, ERβ and AR staining did not vary across different regions (Fig. 2A and Supplementary Fig. 2B). Moreover, there were moderate levels of Ki67 and ERα staining in the central region of AP tumors and in tumors in the dorsal, lateral and ventral prostate (DLVP), but few cells stained in benign areas of the DLVP (Fig. 2A and Supplementary Fig. 2B). Both the differences in Ki67 and ERα positive cells between regions (Supplementary Fig. 2C and 2D) and the correlation between the proportion of Ki67 and ERα stained cells within the prostates of PTEN-deficient mice (Fig. 2B) were statistically significant. The strong correlation between ERα and Ki67 staining implied that ERα may regulate the proliferation of prostate cancer cells.

### ERα regulates proliferation

**Figure 3:**
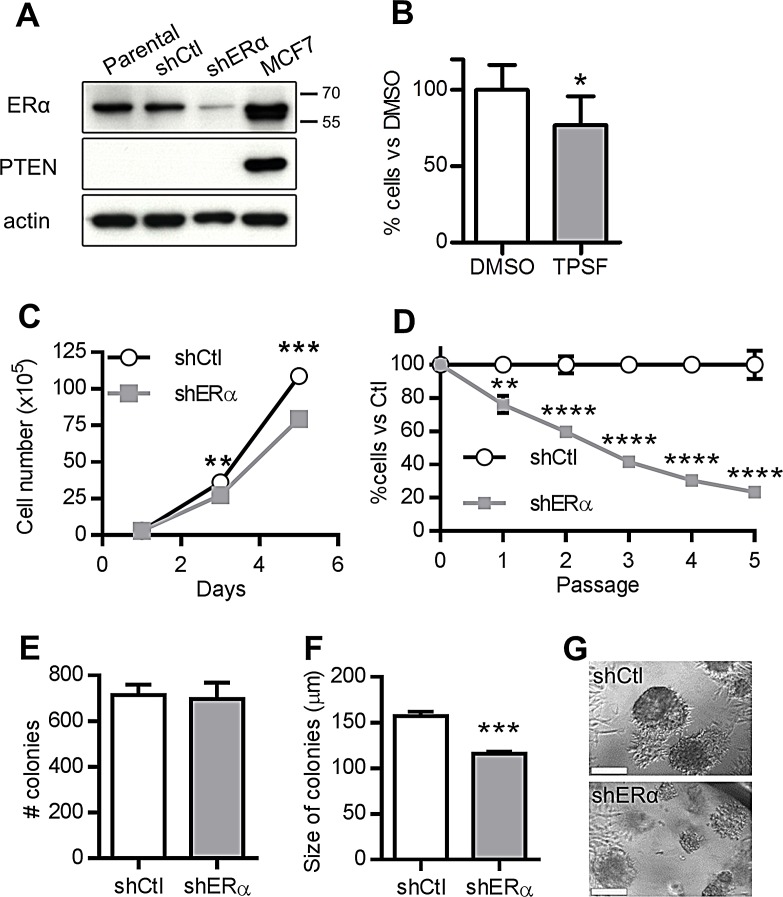
ERα knockdown inhibits the growth of PTEN-deficient prostate cancer cells (A) Representative Western blots of ERα, PTEN and pan-actin levels in parental (untransfected) PTEN-deficient prostate cancer cells and cells stably expressing non-silencing shRNA control (shCtl) or ERα shRNA (shERα). MCF7 cells were used as a control for ERα and PTEN expression. (B) Relative percentage of cells after 5 days of treatment with DMSO or 1 μM TPSF (*P<0.05, paired T Test, n=3). (C) Average number of shCtl and shERα cells after 1, 3 and 5 days of culture (**P<0.01, ***P < 0.001, 2 Way ANOVA, n=3). (D) Average percentage of cells relative to the shCtl over 5 passages (**P<0.01, ****P<0.0001, n=3 flasks, two way ANOVA)^.^ Average number (E) and diameter (F) of colonies formed in Matrigel (***P<0.0001, t test, n=10 wells). (G) Representative phase contrast images of colonies formed by shCtl and shERα knockout cells. Scale bars = 100 μm. Data for shCtl is shown in white and data for shERα is shown in grey.

We investigated whether ERα regulates proliferation using prostate cancer cells derived from the prostate of PTEN-deficient mice. These cells are epithelial, based on the expression of epithelial rather than stromal phenotypic markers (Supplementary Fig. 3A and 3B). They also maintain AR expression (Supplementary Fig. 4A). Immunoblots confirmed that the PTEN-deficient cells also express abundant levels of full length ERα (ERα66) (Fig. 3A, lane 1) like MCF7 breast carcinoma cells, which were used as a positive control (Fig. 3A, lane 4). Treating the PTEN-deficient cells for 5 days with 1 μM of TPSF, a non-competitive antagonist of ERα, reduced proliferation by ~25% (Fig. 3B), similar to its effect on MCF7 cells [34]. There was no change in cell viability, showing that TPSF did not induce apoptosis (Supplementary Fig. 5). To confirm this finding, we also established PTEN-deficient cell lines stably expressing a non-targeting shRNA (shCtl) or an ERα-specific shRNA (shERα). The shCtl cells expressed a similar amount of ERα compared to parental PTEN-deficient cells, while the shERα cells had a ~70% reduction in the amount of ERα (Fig. 3A, lane 2 and 3 and Supplementary Fig. 4B). We then compared the proliferation of shCtrl cells with shERα cells. Consistent with the effect of 1 μM TPSF treatment, ERα knockdown led to a 27% decrease in the number of cells over 5 days (Fig. 3C). Strikingly, the impact of lowering the amount of ERα in PTEN-deficient cells lead to a sustained reduction in the proliferation, demonstrated by a 76.5% decrease in the number of shERα cells compared to shCtrl cells over 5 passages (Fig. 3D). Once again, there was no difference in the cell viability (Supplementary Fig. 5). PTEN-deficient cells form colonies in Matrigel [35]; the ability of individual cells to form colonies is a measure of clonogenicity, whereas the size of each colony indicates their proliferative potential. Depletion of ERα did not change the number of colonies (Fig. 3E), but it led to a significant decrease in the size of the colonies (Fig. 3F and G). This confirms that ERα regulates proliferation of prostate cancer cells.

### ERα sustains PI3K and MAPK signaling

**Figure 4:**
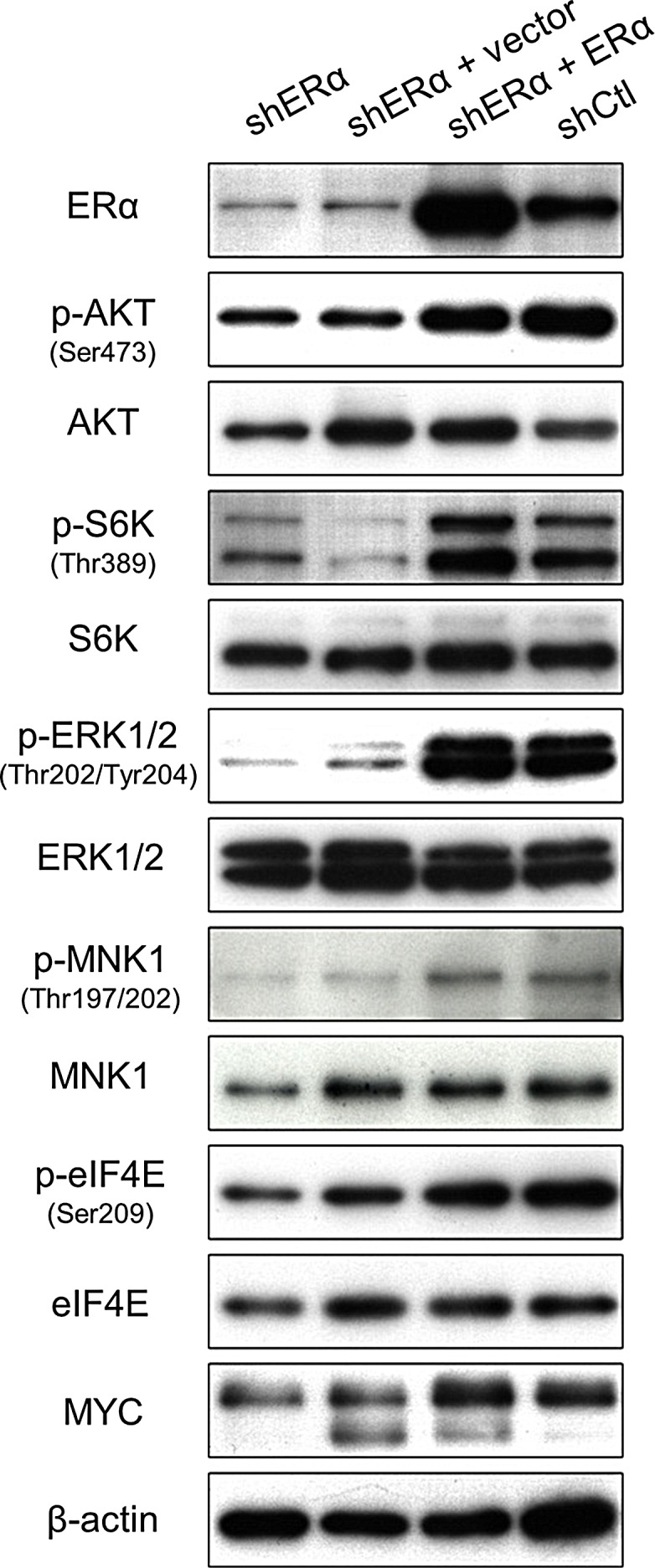
ERα regulates the activity of PI3K and MAPK signaling Representative Western blots of components of the PI3K and MAPK signaling pathways in ERα knockdown (shERα) and control (shCtl) PTEN-deficient cells. The shERα cells were also transfected with an empty vector (+ vector) or full length ERα construct (+ ERα) for 3 days.

Since ERα stimulates PI3K and MAPK signaling, we hypothesized that the decrease in proliferation upon depletion of ERα was due to attenuated activity through these pathways. Therefore, we tested the phosphorylation status of key proteins in extracts from shCtrl cells, shERα cells and, to confirm that any changes in phosphorylation were dependent on ERα expression, shERα cells rescued by transfection with a shRNA-resistant ERα construct. The phosphorylation of AKT (mTORC2 site S473) and S6K (mTORC1 site T389), which are key proteins in the PI3K pathway, was reduced by ERα knockdown, demonstrating attenuated activity downstream of PI3K (Fig. 4). MAPK activity, assessed by phosphorylation of ERK1/2, MNK1 and eIF4E, was also dramatically inhibited by ERα depletion, as indicated by a substantial decrease in pERK1/2 and pMNK1 levels (Fig. 4). MYC is a critical regulator of cell proliferation and survival and its expression is regulated downstream of the PI3K and MAPK pathways which converge on eIF4E [36]. Accordingly, a decrease in MYC protein levels was observed in shERα cells (Fig. 4). Re-expression of ERα rescued PI3K and MAPK activity and restored MYC expression to the levels detected in shCtrl cells, confirming the specific action of ERα. These data demonstrate that ERα sustains the activity of the PI3K and MAPK pathways in prostate cancer cells.

### ERα transcriptome is enriched in metabolic genes

The ERα transcriptome varies between cell types and tissues [37]. As little is known about the transcriptional effects of ERα in prostate epithelial cells, we used DNA microarrays to compare the mRNA abundance in shCtl and shERα cells. Analyses revealed that 1254 genes were up-regulated and 1078 were down-regulated following depletion of ERα (fold-change >1.5 and False Discovery Rate [FDR]<0.1). To assess the functional impact of these vast changes in steady state mRNA levels, we used a gene set enrichment approach to identify prominent functions among differentially regulated genes. Following filtering (FDR<0.15), 5 pathways, all involved in cell division, were up-regulated in shERα versus shCtl cells (Supplementary Fig. 6). In contrast to the relatively few pathways identified among genes that are up-regulated in shERa cells, 51 pathways were enriched among those genes that were down-regulated. Notably, there was an overwhelming abundance of genes involved in carbohydrate metabolism (Table 1), suggesting that ERα modulates such processes either directly or indirectly.

**Table 1:** Down-regulated gene categories in shERα cells related to small molecule metabolism

p.val	q.val	Cellular functions
7.95E-06	0.010	cellular amino acid metabolic process
1.22E-05	0.011	mitochondrial inner membrane
2.29E-05	0.015	small molecule biosynthetic process
5.37E-05	0.029	carbon-carbon lyase activity
0.000324	0.081	sterol biosynthetic process
0.000351	0.081	deoxyribonucleotide catabolic process
0.000423	0.095	glycosaminoglycan biosynthetic process
0.000462	0.099	chondroitin sulfate biosynthetic process
0.000477	0.099	glycosaminoglycan metabolic process
0.000546	0.110	coenzyme metabolic process
0.000588	0.114	deoxyribose phosphate catabolic process
0.000653	0.116	single-organism carbohydrate catabolic process
0.000690	0.116	cofactor metabolic process
0.000691	0.116	hexose catabolic process
0.000778	0.120	NADP metabolic process
0.000787	0.120	proteoglycan biosynthetic process
0.000872	0.125	monosaccharide catabolic process
0.000978	0.128	generation of precursor metabolites and energy
0.001112	0.134	carbohydrate catabolic process

### ERα mediates sensitivity to glucose availability

**Figure 5:**
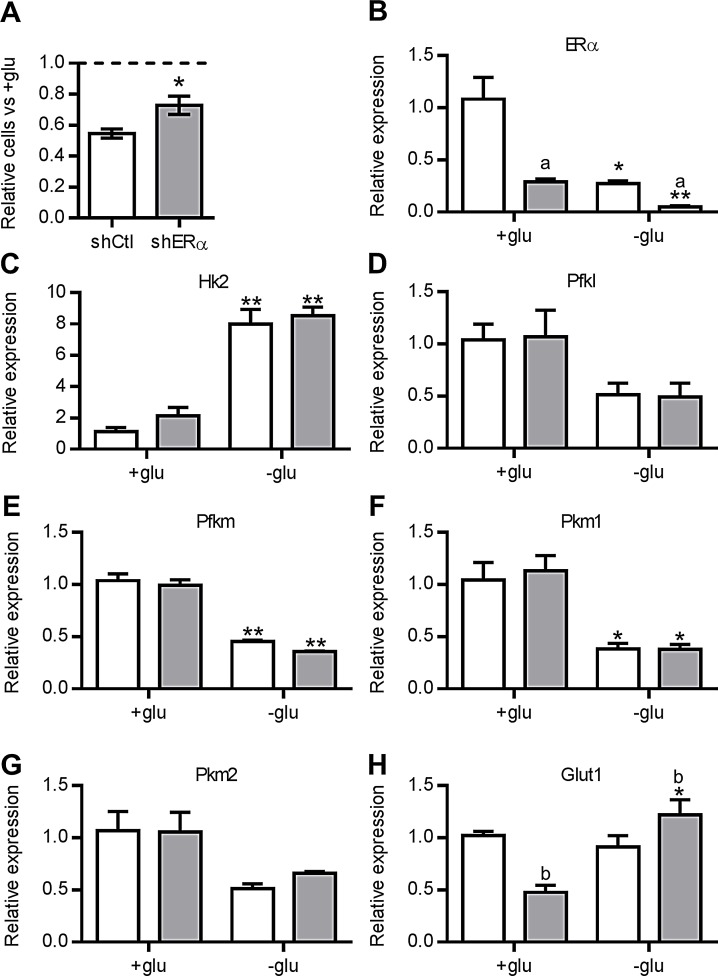
ERα mediates sensitivity to glucose availability (A) Relative number of cells in shCtl (white) and shERα (grey) cells cultured in glucose-free media for 3 days. Data represents relative numbers of cells compared to media with glucose, represented by the dotted line (n=3, *P<0.05, T test). (B-G) Relative gene expression in shCtl (white) and shERα (grey) cells in cultured in basal (+glu) or glucose-free media for 3 days. Data represent relative gene expression normalized to shCtl cells in basal media (n=3, ^a^FDR<0.05, ^b^FDR<0.01 for effect of shRNA; *FDR<0.05, **FDR<0.01, 1 way post-test for the effect of glucose on each shRNA).

PTEN loss has been linked to resistance to caloric restriction *in vivo* [38] and increased glycolysis *in vitro* [39]. In addition, ERα stimulates glucose metabolism in breast cancer cells [40]. Therefore, we tested the impact of altering glucose concentration on the proliferation of shERα cells compared to shCtrl cells. The proliferation of shCtrl cells over three days in the absence of glucose was nearly halved (45.4% decrease) compared to shCtrl cells grown in high glucose medium (Fig. 5A). In contrast, shERα cells were relatively less affected by the absence of glucose, displaying a 27.2% decrease in proliferation compared to shERα cells grown in high glucose medium. This difference in response to glucose withdrawal (*P*<0.05) demonstrates that shCtrl cells are more dependent on glucose availability for proliferation compared to shERα cells, presenting a potential mechanism whereby ERα over-expression may facilitate advanced prostate cancer growth. It is noteworthy that glucose deprivation led to a reduction in ERα mRNA compared to normal glucose conditions; however, the relative levels of ERα in shCtl versus shERα cells were maintained with a 70-80% decrease in shERα cells (Fig. 5B).

Recently, a high throughput screen identified metabolic enzymes and small molecule transporters that confer glucose sensitivity to cancer cells grown in high or low glucose concentrations [41]. The factors giving a proliferative advantage in high glucose concentration were predominantly glycolytic enzymes. Therefore, we examined the relative expression of key enzymes catalyzing the three irreversible steps of glycolysis in shCtl and shERα cells (Fig. 5C-G). There was no significant difference in the expression of hexokinase 2 (*Hk2*), phosphofructokinase l (*Pfkl*) and m (*Pfkm*), or pyruvate kinase isoforms (*Pkm1*, *Pkm2*) between shCtrl and shERα cells grown in high glucose. After glucose deprivation, the relative expression of *Pfkl*, *Pfkm* and *Pkm* isoforms was reduced (Fig. 5D-G), whereas the expression of *Hk2* was increased 4-fold in both shCtrl and shERα cells, suggesting the cells are compensating by increasing hexose phosphorylation activity.

In contrast to the glycolytic enzymes, the glucose transporter *Glut1* (*Slc2a1*) was differentially regulated between shCtrl and shERα cells, consistent with the microarray analysis. *Glut1* was significantly up-regulated in shERα cells deprived of glucose (Fig. 5H), but unchanged in shCtrl cells. Since GLUT1 is the only small molecule transporter demonstrated to affect proliferation of cancer cells in low glucose [41], this striking difference in the regulation of *Glut1* upon glucose deprivation could explain the lesser impact of glucose withdrawal on the proliferation of shERα cells. Collectively, these data show that ERα affects the sensitivity of prostate cancer cells to glucose withdrawal.

## DISCUSSION

The autonomous role of ERα in prostate cancer progression has largely been overlooked. This is due, in part, to confusion about the expression of ERα in prostate cancer. Previous immunohistochemistry studies reported differing levels of ERα in human prostate cancer cells [11-17], possibly due to differences in the range of Gleason score tumors that were examined. We selected a cohort of Gleason score 6 and 9 tumors to ensure that low and high grade tumors were both represented. ERα was expressed in 48% of Gleason score 9 specimens, similar to an early study that detected ERα in 43-61% of Gleason grade 4-5 samples [11]. Another recent study reported that 15% of locally invasive tumours, spanning Gleason score 6 to 10, expressed ERα and that it was significantly associated with biochemical recurrence, decreased progression free survival and poor overall survival [17]. In this study, we also showed that ERα is highly expressed in two different mouse models of aggressive prostate cancer, PTEN-deficient and Hi-MYC mice, mirroring human disease. Together with the estrogenic gene expression signature reported by others [22], this confirms that ERα is indeed expressed in a subset of high grade prostate cancer.

It has long been difficult to study the autonomous actions of ERα in prostate cancer cells because of a paucity of experimental models. Fortunately, our data reveals that ERα is expressed in mouse PTEN-deficient prostate cancer cells both *in vivo* and *in vitro*, making it a suitable model to study the role of ERα during prostate tumorigenesis and progression. Most human cell lines in contrast, express little or no ERα, just as some lack the AR. NCI-H660 cells and some batches of PC3 cells express ERα [18-22], but are not ideal models because they lack AR and have a small cell rather than adenocarcinoma phenotype [42, 43]. Therefore, identifying new ERα human prostate cancer cell lines is a priority. Nevertheless, the ERα negative human prostate cancer cell lines have been useful in studying the mechanisms underlying ERα expression. The lack of ERα expression is due to hypermethylation of the *ESR1* gene, rather than mutation or deletion [19-21]. This suggests that differential methylation could also underlie the re-activation of ERα expression in high grade human prostate cancer tissue. Since tumours with *TMPRSS2-ERG* fusions have an estrogen-regulated gene signature, it is also possible that re-activation of ERα expression is more common in this subset of tumours [22].

Prostate cancer is often considered to be slow growing and to have a low proliferative index, compared to other types of tumors. This is true of most cases of low to moderate grade disease, but a subset of high grade tumors are highly proliferative. Indeed, the percentage of Ki67 positive cells is a powerful prognostic indicator of poor clinical outcome [44]. The significant correlation between Ki67 and ERα staining in PTEN-deficient mice links ERα to rapidly proliferating, aggressive tumors. Importantly, ERα not only correlated with proliferation, it regulated the proliferation of PTEN-deficient prostate cancer cells. This is consistent with a previous study showing that an ERα agonist stimulates the proliferation of a small cell prostate cancer cell line, NCI-H660 [22]. Unexpectedly, even though ERα knockdown reduces the proliferation of PTEN-deficient cells, gene-set enrichment analyses showed that some pathways involved in the M phase were up-regulated. This may represent a compensatory response, albeit insufficient, to the overall decrease in proliferation. Nevertheless, since ERα promotes the proliferation of prostate cancer cells, specifically inhibiting ERα activity could be used to attenuate the growth of a subset of aggressive tumors.

Loss of PTEN is associated with progression to the most aggressive stages of prostate cancer [45]. Initial loss of one allele can be followed by complete lack of expression [29]. Loss of PTEN synergizes with loss of p53, overexpression of MYC and sustained activation of MAPK in promoting prostate cancer initiation and progression [30-32]. Our results demonstrate that ERα can regulate MYC expression upstream of the MAPK pathway. Considering that activating mutations in the RAS/RAF/MEK/ERK signaling cascade are rare in prostate cancer [46, 47], sustained stimulation of the MAPK pathway by ERα is a potential mechanism to increase MYC expression in a subset of prostate cancers.

ERα affects glucose metabolism in human breast cancer cells [40] and our data demonstrate that the same is true in mouse PTEN-deficient prostate cancer cells. Indeed, ERα knockdown decreased the expression of Glut1 (SLC2A1). Highly proliferative PTEN-deficient tumour cells are often sensitive to decreases in glucose levels because they exhibit “glucose-addiction”. In our model, this was attenuated by ERα knockdown because the proliferation of shERα cells was less affected by glucose withdrawal than shCtrl cells. The corollary is that PTEN-deficient prostate cancer cells expressing ERα are reliant on abundant glucose for their rapid proliferation.

In conclusion, we demonstrate that when ERα is expressed in prostate cancer cells it regulates proliferation, MYC expression and glucose sensitivity. The balance between glycolysis and OXPHOS in cancer cells has emerged as a major determinant of sensitivity to antidiabetic biguanides [41]. This is potentially all the more important considering that men taking metformin showed reduced prostate cancer specific mortality [48]. Further work will be required to determine the contribution of classical genomic and rapid non-genomic ERα activities, and the specific contributions of ERα isoforms (66 and 46kDa splice variants), in regulating energy metabolism.

## METHODS

### Patient Specimens

Samples of formalin-fixed and paraffin embedded human prostate tissue, from 36 patients that had undergone radical prostatectomy, were obtained from the Australian Prostate Cancer BioResource with human ethics approval from ethics committees at Epworth Hospital (35906), Cabrini Hospital (10-13-12-04) and Monash University (2005-442). An experienced Uropathologist (J.P.) determined the Gleason Score and percentage of tumor cells expressing ERα in each specimen. Positive ERα staining of tumor cells was defined as prominent nuclear immunoreactivity compared to negative ERα staining in nearby benign glands.

### Animals

ARR2/Pbsn-*Myc* (Hi-MYC) mice were obtained from NCI-Frederick, VA, USA and housed and humanely killed according to Monash University animal ethics approval. The lateral prostate was harvested from 32 week old Hi-MYC mouse or age-matched control mice. PBi-Cre transgenic mice [49] were crossed with *Pten* floxed animals [50] to specifically deplete *Pten* within the prostate epithelium. Animals were maintained on a mixed 129:fvb/n background. *Pten*-deficient (*PBiCre^+/−^;Pten^fl/fl^*) and control mice (*PBiCre^+/−^*) were used at 80-100 d of age for all studies. Experiments were performed according to animal ethics approval from the Peter MacCallum Cancer Centre.

### Immunohistochemistry

Immunohistochemistry was performed manually with the DAKO EnVision+ detection system or with an autostainer using the Leica BOND-MAX system (Leica Microsystems Pty. Ltd.). The concentration and specific conditions for each primary antibody are listed in Supplementary Table 2. The specificity of the ERα antibody has been confirmed by several independent laboratories [11, 12, 51].

### Stereology

Slides of stained tissues were imaged with an Aperio ScanScope digital scanner and viewed using ImageScope software (Aperio). The dorsal, lateral and ventral lobes were divided into benign and malignant foci. Within the anterior prostate, tumor foci were divided into peripheral tumor regions, within 50 μm of the stroma, and central tumor regions greater than 100 μm from the stroma. The percentage of ERα or Ki67 positive cells was manually counted in at least 3 fields per region (average ~10 fields) for 3 mice. Each field contained an average of approximately 150 cells. Data is represented as the average percentage of positively stained cells in each region per mouse.

### Cell Culture

MCF7 cells were purchase from the American Type Culture Collection and cultured in RPMI medium containing 5% fetal bovine serum (FBS), 100 IU/ml penicillin and 10 μg/ml streptomycin (Invitrogen). An epithelial prostate cancer cell line was established from an invasive prostate carcinoma harvested from a 407 day old male *PBiCre^+/−^;Pten^fl/fl^* mouse. Briefly, prostate tumor sections approximately 5 mm^3^ were placed on ice for 1 hr in PBS supplemented with 3% penicillin streptomyocin (Pen Strep, Gibco) prior to homogenization. Samples were then transferred to a T25 flask with 5 ml of α-MEM medium containing 20% FBS, 1% soduim pyruvate, 80 μg/ml Fluconazole, 1% Pen Strep and 1% Glutamate (Gibco). Media was replaced every 2-3 days. Cultures were then maintained in high glucose DMEM medium containing 10% FBS, 100 IU/ml penicillin and 10 μg/ml streptomycin (Invitrogen) and kept in a humidified incubator at 37°C with 5% CO_2_.

### Estrogen antagonist treatments

PTEN-deficient prostate cancer cells were seeded in 6 well plates (2 × 10^4^/well) and cultured for 24 h. Cells were then treated daily for 4 days with media containing DMSO or 1 μM TPSF (p-fluoro-4-(1,2,3,6,-tetrahydro-1,3-dimethyl-2-oxo-6-thionpurin-8-ylthio). On the fifth day, cells were trypsinized, resuspended in media and counted using a TC10 automated cell counter (BioRad). The experiment was performed three times in triplicate.

### ERα Knockdown

Knockdown experiments were performed using the pGIPZ vector (Open Biosystems) containing either a non-silencing control shRNA or a mouse ERα shRNA (V2LMM_30677). Viral stocks were prepared from 293-T cells using the Trans-Lentiviral shRNA Packaging kit according to the manufacturer's instructions. PTEN-deficient cells were transduced, sorted for GFP expression and then maintained in medium supplemented with 4 μg/mL puromycin.

### Proliferation Assays

To assess proliferation, 2 × 10^4^ cells were seeded per well in 6-well plates and cultured for 1, 3 or 5 days before being trypsinized and counted using a TC10 automated cell counter (BioRad). For all cell counts, trypan blue was used to measure cell viability, which was typically between 97-100% and was not affected by any treatment. Therefore, all data are reported as numbers of viable cells. Cumulative differences in proliferation were measured by seeding, counting and re-seeding 5 × 10^4^ cells every 4 days for 5 passages. The proliferation of cells in three dimensional conditions was measured by seeding 3000 cells in 100 μL of a 2:3 mixture of media to growth factor-reduced Matrigel (BD Biosciences). The solution was set around the edge of the wells of a 12-well plate and the cells were cultured for 10 days. The number of colonies was counted with a phase contrast microscope at 20 times magnification. The size of colonies was measured using ImageJ with images taken at 4 positions per well, typically containing a total of 20-30 colonies.

### ERα Rescue

To rescue full length ERα expression, 1 × 10^5^ PTEN-deficient cells were seeded per well in 6 well plates for 24 hr and then transfected with 2.5 μg of a human ERα66 construct (pSG5-HEGO) or empty vector control using 12.5 μl/well of lipofectamine 2000. Cells were harvested after 72 hrs.

### Western blotting

Samples were lysed in RIPA buffer containing 5 mM NaF, 1 mM Na_3_VO_4_ and 1x complete protease inhibitor cocktail (Roche) and quantified using the bicinchoninic acid assay (Promega). Lysates were separated using polyacrylamide gel electrophoresis and probed with primary antibodies listed in Supplementary Table 1. Membranes were then incubated with HRP-conjugated secondary antibodies (goat anti-rabbit, goat anti-mouse (Dako), goat anti-rat (Cell Signaling)) followed by ECL Plus Western Blotting Detection Reagent (GE Healthcare).

### Microarrays

Cells were lysed in hypotonic lysis buffer (5 mM Tris-HCl [pH7.5], 2.5 mM MgCl2, 1.5 mM KCl, 100 μg/ml cycloheximide, 2 mM DTT, 0.5% Triton X-100, and 0.5% sodium deoxycholate), supernatant was cleared by centrifugation and total RNA was isolated using TRIzol Reagent (Life Technologies). RNA from biological triplicate experiments were labelled according to the manufacturer's guidelines (Affymetrix), hybridized to the Affymetrix Gene ST 1.1 Gene Titan array and scanned using the Affymetrix GeneTitan scanner. The resulting .CEL files were normalized using updated probe set definitions [52], because these provide improved precision and accuracy as compared to the default probe set definitions [53], and the robust multi-array average (RMA) algorithm [54] using the “affy” R (r-project.org) package. Differential expression was identified using the random variance model t-statistics [55] and the resulting p-values were adjusted using the Benjamini-Hochberg False Discovery Rate (FDR) method [56]. Genes with an FDR<0.1 and a fold-change >log2(1.5) were considered significantly differentially expressed. To identify functions enriched among genes at the ends of a gene list ranked by signed (up[+] or down[−]) –log10(p-values), we used GAGE [57] and annotations from the gene ontology consortium [58]. Within the “gage” function we used rank.test=TRUE and default settings (i.e. categories containing between 10 and 500 genes were considered). Categories with an FDR<0.1 were considered significant enriched.

### Glucose Deprivation

Cells were seeded at 2 × 10^4^ cells per well in 6 well plates in standard high glucose (25 mM) DMEM medium containing 10% FBS, 100 IU/ml penicillin and 10 μg/ml streptomycin (Invitrogen). After 24 hr, the medium was changed to standard high glucose medium or glucose-free DMEM, both containing 10% FBS and antibiotics. After 3 days, the cells were trypsinized and counted using an automated cell counter. Data was normalized to the average number of cells in high glucose medium. The experiment was repeated 4 times in triplicate.

### RNA extraction and quantitative RT-PCR

Total RNA was extracted using an RNeasy kit (Qiagen) and treated with DNase I according to the manufacturer's instructions (Life Technologies). Complementary DNA was synthesized using the SuperScript III First Strand Synthesis System (Life Technologies). Samples were amplified using Power Sybr Green (Life Technologies) and gene-specific primers (Sigma Aldrich; Supplementary Table 3) on an ABI7900 thermocycler (Life Technologies). Target genes were normalized to the geometric mean of three reference genes, RPLP0, HPRT1 and UBC. Relative gene expression was calculated using the delta delta Ct method.

### Statistical Analyses

GraphPad Prism software was used to generate graphs and perform statistical analyses, which are noted in figure legends. All data are displayed with standard error of the mean calculated from independent experiments.

## SUPPLEMENTARY, TABLES AND FIGURES


