# Supplementary material for: Estrogen receptor alpha drives proliferation in PTEN-deficient prostate carcinoma by stimulating survival signaling, MYC expression and altering glucose sensitivity
Source: Oncotarget. 2014 Nov 26;6(2):604–16. doi: 10.18632/oncotarget.2820 (PMC4359242; doi:10.18632/oncotarget.2820)
Supplement: Supplementary file 1 [file oncotarget-06-604-s001.pdf]

Estrogen receptor alpha drives proliferation in PTEN-deficient prostate carcinoma by stimulating survival signaling, MYC expression and altering glucose sensitivity

Supplementary Material

Supplemental Table 1
Percentage of cancer epithelial cells expressing ERα in human prostate cancer specimens

Gleason Score	ERα#	AR
6	0	90
6	0	N.D.
6	0	100
6	0	100
6	0	100
6	0	100
6	0	100
6	0	90
6	0	N.D.
6	0	90
6	0	90
6	0	70
6	0	100
6	0	90
6	0	90
9	**70**	100
9	**80**	100
9	**20**	100
9	0	80
9	0	N.D.
9	0	N.D.
9	**10**	90
9	**10**	90
9	**<10**	90
9	0	90
9	0	90
9	**80**	90
9	0	90
9	0	90
9	0	90
9	0	90
9	0	80
9	0	80
9	**60**	90
9	**30**	90
9	**30**	90

N.D = Not detected due to poor staining or lack of tumour cells in the section
#*P*=0.001 significant correlation (r=0.524) between ERα positivity and Gleason score and *P*=0.002 significant correlation (r=0.502) between percentage of ERα positive cells and Gleason score (Spearman test, n=36).

No significant correlation between AR expression and Gleason score.

Supplemental Table 2
Primary antibodies used for immunohistochemistry (IHC) and western blotting (WB).

**Antigen**	**Isotype**	**Clone/ID**	**Supplier**	**IHC concentration and conditions**	**WB concentration**
Ki67	Mouse IgG1	MM1	Novocastra	1:400[(A)(2)]	-
ERα	Mouse IgG1	6F11	Novocastra	Mouse 1:300[(A)(2)]<br>Human 1:100[(A)(3)]	1:200
ERβ	Mouse IgG1	EMR02	Novocastra	1:300[(A)(3)]	-
AR	Rabbit polyclonal	#A9853	Sigma Aldrich	2 μg/mL[(C)(4)]	-
PTEN	Rabbit IgG	138G6	Cell Signalling Technology	1:100[(B)(1)]	1:1000
Phospho-Akt (Ser473)	Rabbit IgG	D9E	Cell Signalling Technology	1:100[(B)(1)]	1:1000
Akt	Rabbit IgG	C67E7	Cell Signalling Technology	-	1:1000
Phospho-p70 S6 Kinase (Thr389)	Rabbit polyclonal	#9205	Cell Signalling Technology	-	1:1000
p70 S6 Kinase	Rabbit IgG	49D7	Cell Signalling Technology	-	1:2500
Phospho-p44/42 MAPK (Erk1/2) (Thr202/Tyr204)	Rabbit IgG	D13.14.4E	Cell Signalling Technology	-	1:1000
p44/42 MAPK (Erk1/2)	Rabbit IgG	137F5	Cell Signalling Technology	-	1:2500
Phospho-Mnk1 (Thr197/202)	Rabbit polyclonal	#2111	Cell Signalling Technology	-	1:1000
Mnk1	Rabbit IgG	C4C1	Cell Signalling Technology	-	1:1000
Phospho-eIF4E (Ser209)	Rabbit IgG	EP2151Y	Abcam	-	1:1000
eIF4E	Rabbit polyclonal	#9742	Cell Signalling Technology	-	1:3000
MYC	Rabbit IgG	Y69	Abcam	-	1:2000
Pan-actin	Rabbit polyclonal	#4968	Cell Signalling Technology	-	1:1000

A – BondTM epitope retrieval 2 (Leica)
B – Microwave antigen retrieval in Tris-EDTA (1 mM) pH 9.0 buffer
C – BondTM epitope retrieval 1 (Leica)
1 – EnVision labelled polymer-HRP anti-rabbit with DAB substrate (DAKO)
2 – EnVision labelled polymer-HRP anti-mouse with DAB substrate (DAKO)
3 – BondRefine Detection kit (Leica)
4 – BondRefine Detection kit with rabbit secondary only (Leica)

Supplemental Table 3
Primers used for quantitative RT-PCR.

Transcript	Forward Primer (5'-3')	Reverse Primer (5'-3')	Ref
RPLPO	GCGTGGCAATCCCTGACGCAC CGCCG	TGCGGATCTGCTGCATCTGCTTGGAGC	
UBC	CGTCGAGCCCAGTGTTACCAC CAAGAAGG	CCCCCATCACACCCAAGAACAAGCACA AG	1
HPRT1	TGGCCGGCAGCGTTTCTGAG	TCGCTAATCACGACGCTGGGAC	
ESR1	GGTCCTGCGAAGGCTGCAAGG	TTCTCCCTCCTCGGCGGTCT	
AR	TTGGACAGTACCAGGGACCA	CTTCTGTTTCCCTTCAGCGG	
SLC2A1	TGGCGGGAGACGCATAGTTA	AACTCCTCAATAACCTTCTGGGG	
HK2	GGCTAGGAGCTACCACACAC	AACTCGCCATGTTCTGTCCC	
PFKL	CGCTGCAATGGAGAGTTGTG	CCTCAAAGACGTAGGCAGCA	
PFKM	ATCGTAGACGCCATCACCAC	GACAAGGGCCAGGTATCCAC	
PKM1	GTCTGGAGAAACAGCCAAGG	TCTTCAAACAGCAGACGGTG	2
PKM2	GTCTGGAGAAACAGCCAAGG	CGGAGTTCCTCGAATAGCTG	2

1. Mamo S, Gal AB, Bodo S, Dinnyes A. Quantitative evaluation and selection of reference genes in mouse oocytes and embryos cultured in vivo and in vitro. *BMC Dev Biol* 2007;**7**: 14.

2. Israelsen WJ, Dayton TL, Davidson SM, Fiske BP, Hosios AM, Bellinger G, Li J, Yu Y, Sasaki M, Horner JW, Burga LN, Xie J, et al. PKM2 isoform-specific deletion reveals a differential requirement for pyruvate kinase in tumor cells. *Cell* 2013;**155**: 397-409.

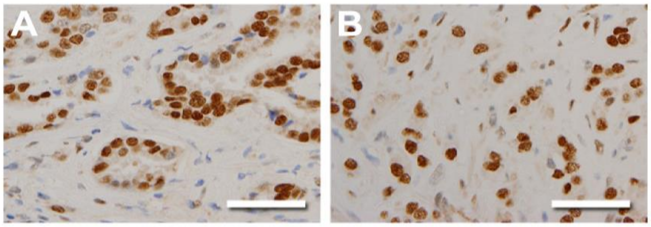

Supplementary Figure 1: AR expression is maintained in human prostate cancer.
Representative images of AR staining in specimens of (A) Gleason score 6 and (B) Gleason score 9 human prostate cancer. Scale bars equal 50 µm.

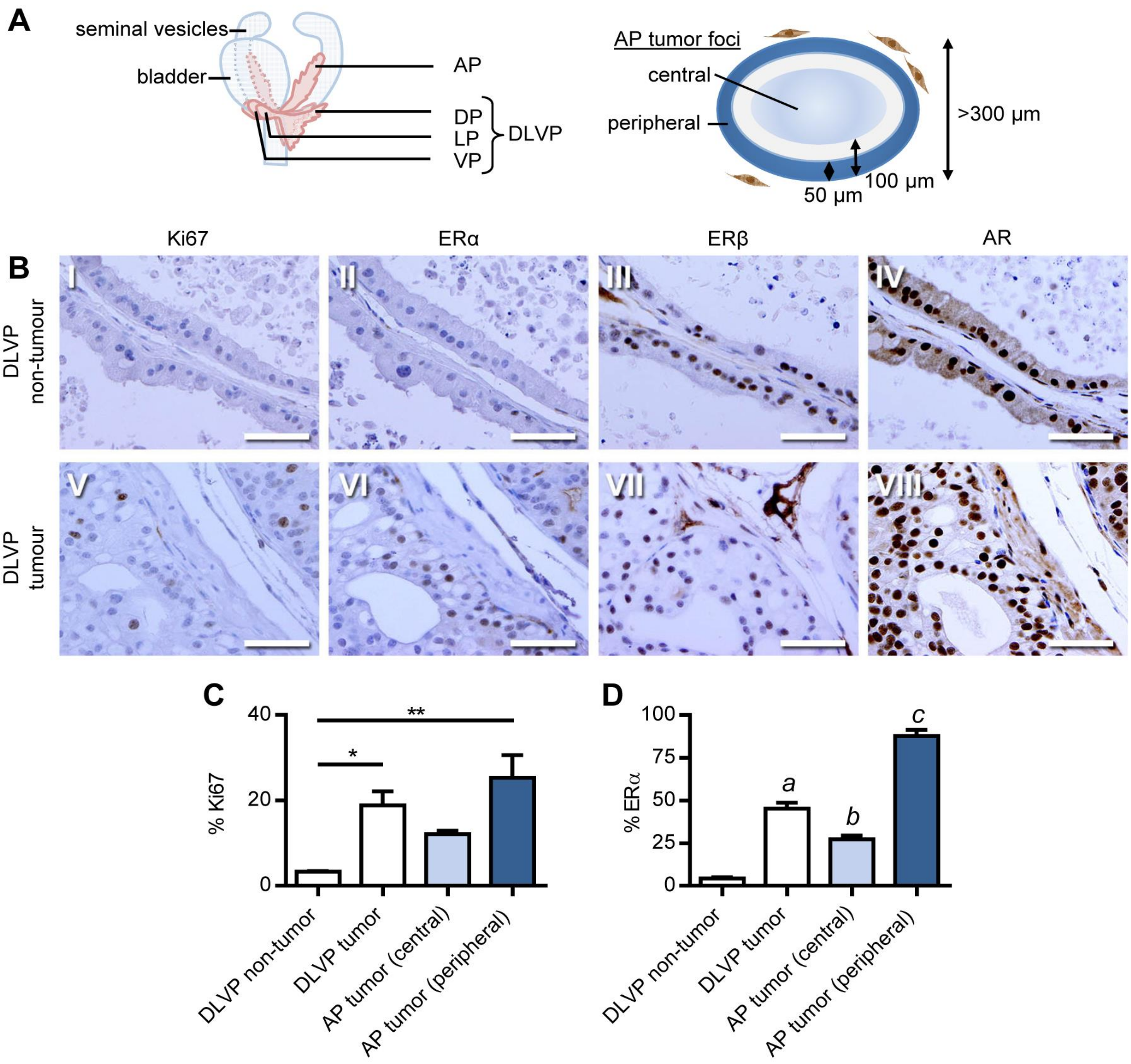

Supplementary Figure 2

Correlation between ERα and Ki67 in PTEN-null prostate cancer

(**A**) Schematic of mouse prostate and definition of central and peripheral regions in anterior prostate (AP) tumors. The dorsal, lateral and ventral prostate (DLVP) was divided into tumor and benign (non-tumor) regions. The AP has large malignant foci, but no benign glands, in 3 month old PTEN-null mice. Therefore, foci were divided into the Ki67-rich peripheral regions, within 50 µm of the stroma, and Ki67-low central regions, greater than 100 µm from the stroma. (**B**) Representative images of immunohistochemical staining of Ki67 (I, V), ERα (II, VI), ERβ (III, VII), and AR (IV, VIII), in DLVP non-tumor (I - IV) and DLVP tumor (V - VIII) regions. Scale bars = 50 µm. Average percentage of Ki67 (**C**) and ERα (**D**) positive cells in different regions of PTEN-null prostates (One way ANOVA, n=3 mice, **P*<0.05; ***P*<0.01; a *P*<0.001 vs DVLP non-tumor, *P*<0.01 vs AP tumor-central; b *P*<0.01 vs DVLP non-tumor; c *P*<0.001 vs all groups).

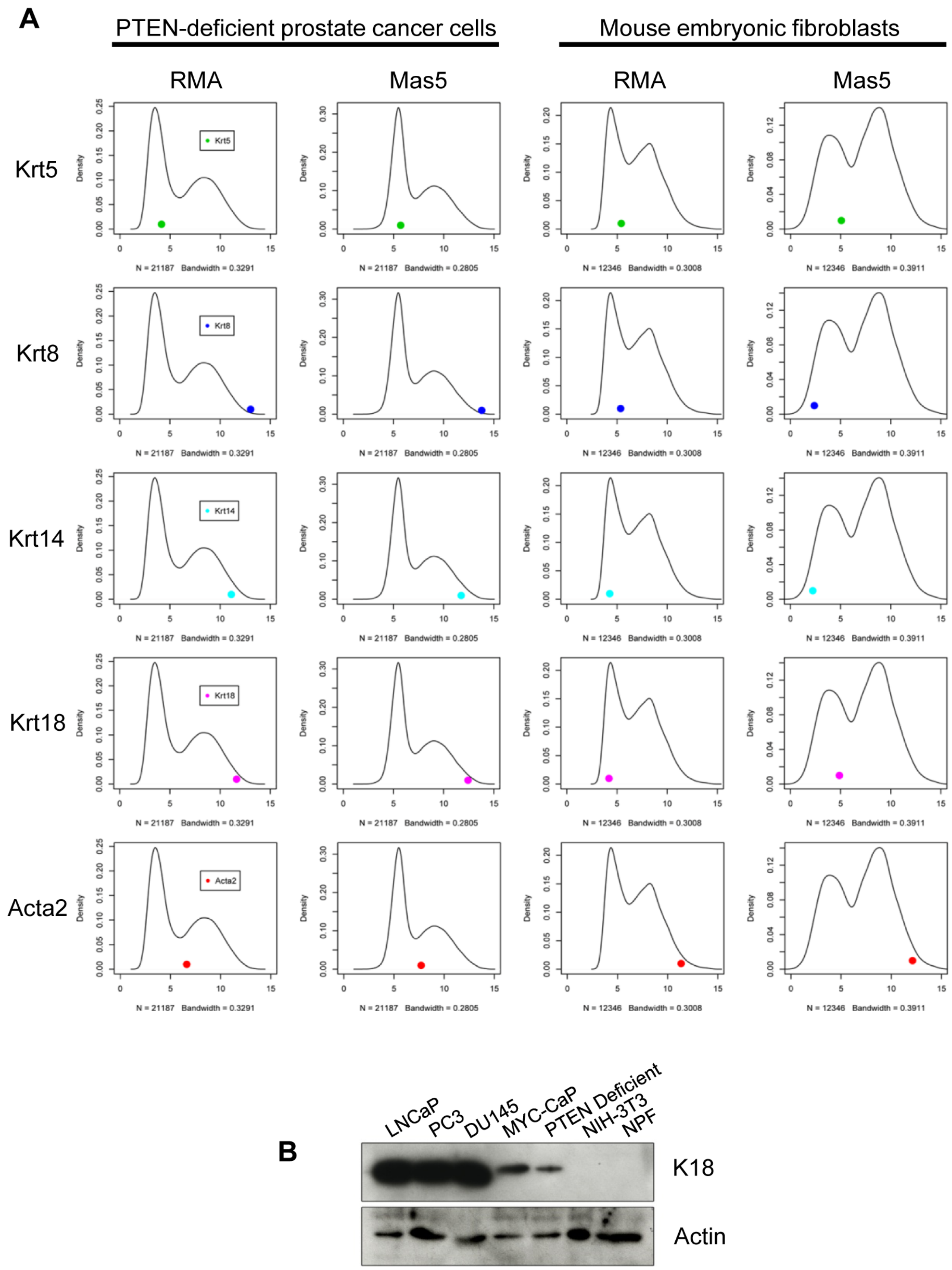
A
PTEN-deficient prostate cancer cells
Mouse embryonic fibroblasts
RMA
Mas5
RMA
Mas5
Krt5
Krt8
Krt14
Krt18
Acta2
Density
N = 21187 Bandwidth = 0.3291
N = 21187 Bandwidth = 0.2805
N = 12346 Bandwidth = 0.3008
N = 12346 Bandwidth = 0.3911
B
LNCaP
PC3
DU145
MYC-CaP
PTEN Deficient
NIH-3T3
NPF
K18
Actin

Supplementary Figure 3: The PTEN-deficient prostate cancer cell line has an epithelial phenotype. (**A**) Comparison of microarray data from the PTEN-deficient prostate cancer cells with a previously published dataset for mouse embryonic fibroblasts carrying a tet-inducible *Pten* transgene (GSE29010). Each dataset was normalised using two approaches, RMA and Mas5. Expression values form two distributions: non-expressed genes (left peak) and expressed genes (right peak). *Krt5* could not be detected in either cell type. *Krt8*, *Krt14* and *Krt18* are expressed in PTEN-deficient prostate cancer cells, but not fibroblasts. *Acta2* is highly expressed in fibroblasts, but not PTEN- deficient prostate cancer cells. (**B**) Western blot for keratin 18 in parental PTEN-deficient cells confirming their epithelial phenotype. Positive controls include human (LNCaP, PC3, DU145) and mouse (MYC-CaP) prostate cancer epithelial cells cells. Negative controls include mouse NIH-3T3 fibroblasts and human normal prostate fibroblasts (NPFs).

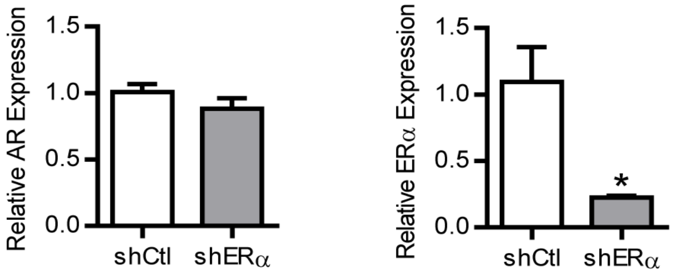

Supplementary Figure 4: Expression of AR and ERα in PTEN-deficient cells.
Relative expression of AR and ERα in shCtl and shERα PTEN deficient cells. Data represent average expression relative to shCtl cells with RPLPO as the reference gene (**P*<0.05, t test, n=3).

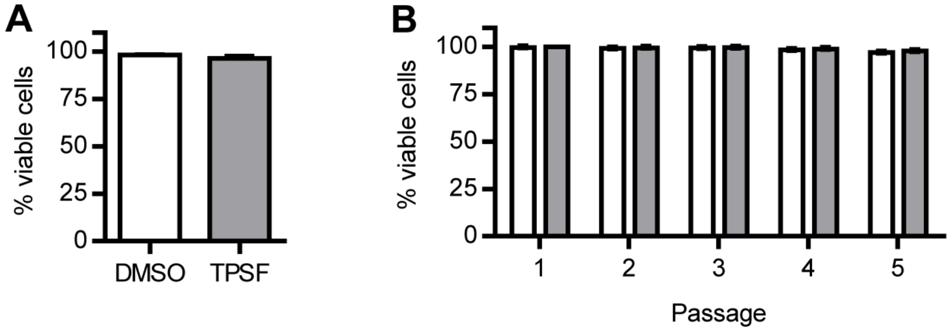

Supplementary Figure 5: Stable knock down of shERα does not affect cell viability.
(**A**) Average percentage of viable cells in parental PTEN-deficient cells treated with DMSO or TPSF. (**B**) Average percentage of viable cells in shCtl (white) and shERα (grey) cells cultured over 5 passages.

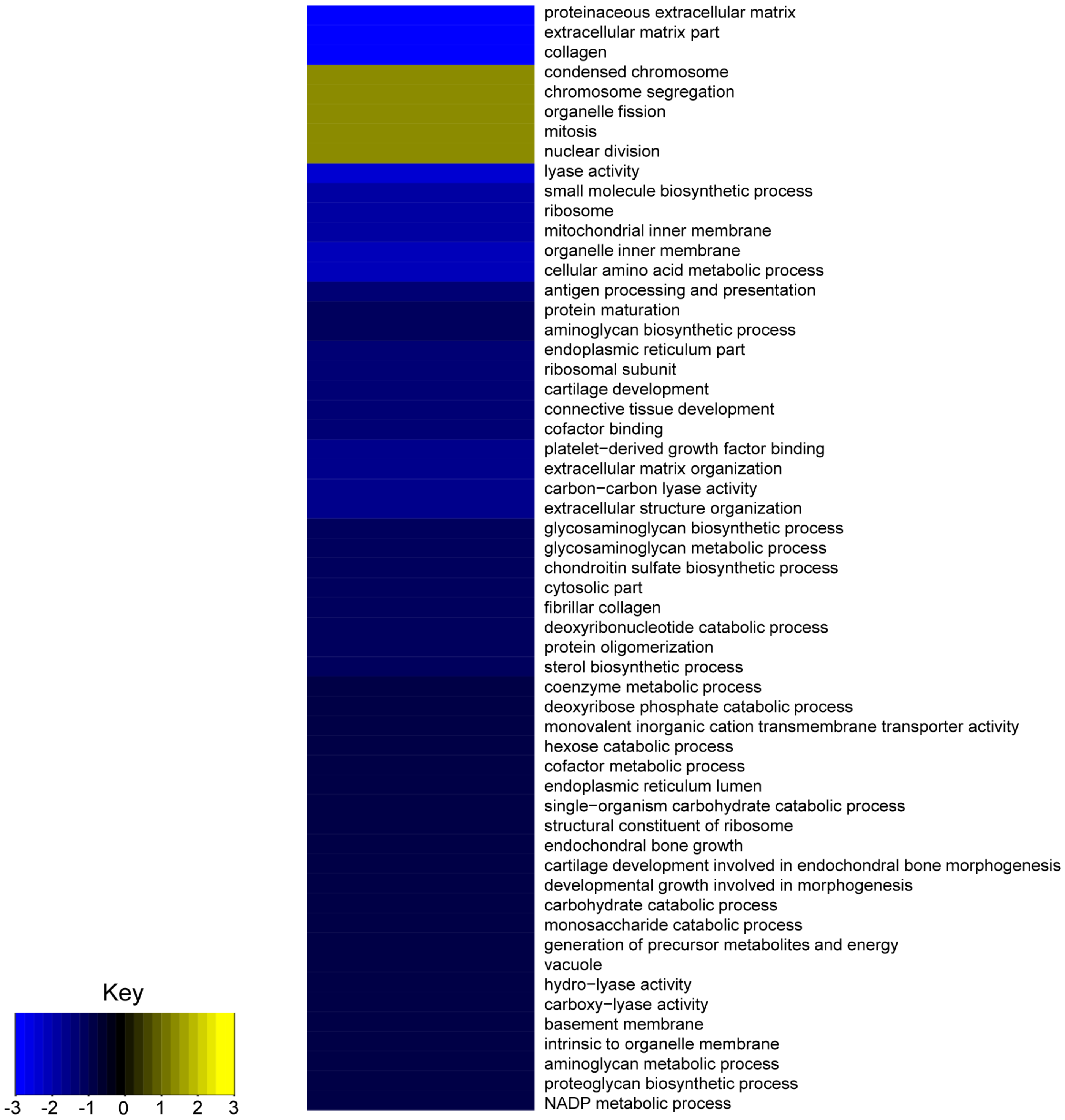

Supplementary Figure 6
Pathway analysis of differentially expressed genes between shCtl and shERα cells

The color code represents -log10(FDR) for enrichment of pathways among up (+ and yellow) or down (- and blue) regulated genes in shERα cells as compared to their control.
